# Institutional Notes and News

**Published:** 1911-02-25

**Authors:** 


### Institutional Notes and News.

**Extending Swinton Hospital.**

At the last general meeting of the Wath, Swinton, and District Joint Hospital Board it was stated that considering the hospital board was contemplating spending £2,000 on hospital extensions it seemed rather peculiar that two nurses were leaving the hospital on account of a reduction of staff. The Medical Officer (Dr. Burman) explained that there were few patients in at that time, but since then scarlet fever had broken out at Swinton. The Local Government Board has sanctioned loans of £1,710 and £290 respectively for additional ward accommodation, subject to the usual building conditions. The recommendation of the House Committee that the contract for the extensions be let to Mr. W. M. Beedon, of Thryberg, at £1,663 14s. 4d., was approved.

**Royal Visit to St. George's Hospital.**

Princess Christian has visited St. George's Hospital to receive an address thanking her for accepting the office of President of the Hospital in succession to the King, and has become patron. Her Royal Highness, in replying to the address, said: "For many years past I have taken the deepest interest in this hospital and watched its successful progress with the keenest interest. That interest will, if possible, be redoubled now, and nothing will be lacking on my part to help on the hospital in every way in my power. Your kind reference to my connection with nursing and nurses is most gratifying to me, as for more than 30 years it has been my great object to raise nursing as a profession to that honourable position it now occupies, and it is no little satisfaction to me to know that I helped to raise the standard of nursing to that which is now practically obligatory." Princess Christian then made a tour of the hospital, visiting most of the wards, the nurses' rooms, and the kitchen.

**Dewsbury King Edward Memorial.**

The question of the provision of a suitable King Edward memorial was considered at a meeting at Dewsbury on Thursday last. The Mayor (Major P. B. Walker) was in favour of attention being given to a suggestion that a sanatorium for consumptives might be provided, and he expressed the opinion that the carrying into effect of the proposal would be in accordance with the wishes and desires of the late King, who took so much interest in the subject of helping the sick and afflicted. Sir Mark Oldroyd, a well-known public benefactor, who was unable to be present, wrote suggesting that certain hospital buildings in the Thornhill District might easily be adapted for use as a sanatorium in connection with the existing Infirmary. Eventually it was resolved that a public fund should be opened, and a committee was appointed to consider various schemes.

**German Hospital, Dalston.**

The Annual General Court of Governors was held at Winchester House, E.C., last week, Baron von Schröder (the Treasurer) in the chair. The report of the committee showed a steady increase in the usefulness and activities of the institution. The ordinary income amounted to £11,614 14s. 5d., and the expenditure to £11,993 6s. 11d. The hospital has had to deplore the death, on April 20 last, of Baron J. H. W. von Schröder, whose resignation of the offices of Trustee and Treasurer of the hospital was referred to in the last report. The late baron bequeathed to the hospital the munificent sum of £10,000, and as a memorial to his generosity and long and active connection with the hospital, it has been decided to name the children's ward "The Baron Schröder Ward." Another old friend of the institution has been lost through the death of Mr. Daniel Meinertzhagen, who for upwards of sixteen years was one of the trustees. In consequence of the death of his late Majesty King Edward VII., the Annual Dinner, which was fixed to take place on May 15 last, under the presidency of the Right Hon. Sir Frank Cavendish Lascelles, was abandoned. The stewards, however, kindly completed their exertions on behalf of the hospital, and succeeded in getting together the splendid amount of £4,151 7s. 7d. This sum is not far short of the collections of the two previous years, which were above the average, and, as the hospital saved the expense of the festival—about £250—the result must be considered extremely satisfactory. The report referred to the building of the new nurses' home, details of which were given in The Hospital, Dec. 31, 1910, page 417. As soon as this work is completed, it is intended to proceed with the other structural alterations and additions which include new balcony wards on the south side of the hospital; new bacteriological and pathological laboratories; two additional operating rooms, one for septic, and one for ophthalmic cases; new lavatories for the staff; a new bathroom for patients upon admission, and before transference to the wards; additional accommodation for the resident medical and dispensing staff; a new passenger-lift; and finally, much needed additions and improvements in the out-patients' department and the mortuary. The contributions to the Special Building Fund now amount to £28,507 19s., but as the improvements indicated will involve a much larger outlay than was at first intended the committee confidently appeals for further assistance.

**Liverpool Royal Infirmary.**

The new out-patient department which is being built at the Liverpool Royal Infirmary is expected to be ready for its formal opening next month; and the need for increased accommodation at the hospital is shown by the increase in the number of applicants for out-patient treatment which is now becoming a familiar item in hospital reports. The out-patient department at Liverpool has been designed to give special facilities for the treatment and the study of eye, ear, nose, and women's diseases. The building fund is however short by £6,000, towards which it is hoped the Corporation may subscribe £5,000, if the necessary powers are obtained. The University has made a grant for bacteriological examinations. The three houses in Ashton Street now occupied by the nurses are expected to include at least one more, so that the night nurses can be placed away from the noise and bustle of the hospital under the charge of a superintendent. The efforts made last year to form a new maintenance fund realised £14,000; if another £11,000 be raised the fund will have recovered the importance and extent which it possessed twenty years ago. Lord Sefton presided at the annual meeting, and announced that he must retire from the office of president of the infirmary, an office which, he recalled with pleasure, had been filled formerly by his grandfather. The Infirmary authorities are still pressing on the Corporation the force of the resolution which they passed in 1908, that the removal of the abattoirs from Trowbridge Street is essential to the welfare of the hospital. The need for this removal, as Mr. Ralph Brocklebank remarked, has been discussed since 1867. It is probable that a decision one way or the other will be arrived at very shortly, as the Corporation is now considering the question.

